# Properties of Antioxidant Film Based on Protein Isolate and Seed Coat Extract from Bambara Groundnut

**DOI:** 10.3390/foods13213424

**Published:** 2024-10-27

**Authors:** Jilmika Kantakul, Krisana Nilsuwan, Chanikarn Kotcharat, Kanokporn Chuecheen, Jirakrit Saetang, Thummanoon Prodpran, Hui Hong, Bin Zhang, Soottawat Benjakul

**Affiliations:** 1 International Center of Excellence in Seafood Science and Innovation (ICE-SSI), Faculty of Agro-Industry, Prince of Songkla University, Hat Yai 90110, Songkhla, Thailand; jilmika.k@gmail.com (J.K.); chanikarn.kr79@gmail.com (C.K.); aimkanokpornaim@gmail.com (K.C.); jirakrit.s@psu.ac.th (J.S.); thummanoon.p@psu.ac.th (T.P.); soottawat.b@psu.ac.th (S.B.); 2 Center of Excellence in Bio-Based Materials and Packaging Innovation, Faculty of Agro-Industry, Prince of Songkla University, Hat Yai 90110, Songkhla, Thailand; 3 Beijing Laboratory for Food Quality and Safety, College of Food Science and Nutritional Engineering, China Agricultural University, Beijing 100083, China; hhong@cau.edu.cn; 4 Key Laboratory of Health Risk Factors for Seafood of Zhejiang Province, College of Food Science and Pharmacy, Zhejiang Ocean University, Zhoushan 316022, China; zhangbin@zjou.edu.cn; 5 Department of Food and Nutrition, Kyung Hee University, Seoul 02447, Republic of Korea

**Keywords:** Bambara groundnut, seed extract, protein isolate, properties

## Abstract

Bambara groundnut (BG)-based films containing seed coat extract at different concentrations were prepared and characterized. BG seed coat extract (BGSCE) had a total phenolic content of 708.38 mg GAE/g dry extract. BGSCE majorly consisted of quercetin 3-galactoside, rutin, and azaleatin 3-arabinoside. BGSCE exhibited ABTS and DPPH radical scavenging activities (ABTS-RSAs and DPPH-RSAs), a ferric reducing antioxidant power (FRAP), and an oxygen radical absorbance capacity (ORAC) of 66.44, 4.98, 4.42, and 0.91 mmol Trolox equivalent/g dry extract, respectively. When BGSCE at various concentrations (0–8%, *w*/*w*, protein content) was incorporated into the BG protein isolate (BG-PI)-based films, film containing 4% BGSCE exhibited higher thickness, tensile strength, elongation at break, water vapor and UV-light barrier properties, and a*-value (redness) than the control film (*p* < 0.05). Films containing BGSCE had greater ABTS-RSA, FRAP, and ORAC than the control film (*p* < 0.05). An FTIR analysis elucidated that the proteins interacted with phenolic compounds in BGSCE. Nonetheless, less thermal stability was attained in films added with BGSCE. Hence, the addition of BGSCE possessing antioxidant activity exhibited an important role in properties and characteristics of BG-PI-based film. The developed active film could be applied as packaging material possessing antioxidant property for food applications.

## 1. Introduction

Food packaging materials are crucial for protecting food products and facilitating their distribution. Petroleum-based materials are prevalent due to their numerous advantages. However, these materials pose significant environmental challenges, as they are not biodegradable and contribute to pollution globally [1]. Proteins are used for making biodegradable films since they are abundant and show effective film-forming capabilities [2]. Protein isolate, especially from plant origins, is a material used for making edible/biodegradable films and coatings since it exhibits outstanding film-forming properties [3]. Legume protein isolates have gained significance in the food industry [4]. Nevertheless, most applications are dominated by soybean, while proteins from other legume proteins are less utilized. Zhao et al. [3] reported that the protein films fabricated from soy protein isolate (SPI) showed high tensile strength (9.23 MPa) and water vapor permeability (2.72 × 10^−10^ g/m s Pa). Bambara groundnut (*Vigna subterranea*) is widely cultivated in various regions globally [5] and serve as the diet rich in protein and amino acids, particularly for people in the southern areas of Thailand. A deeper understanding of legume protein isolates can facilitate their effective applications. Furthermore, the plant extracts have been employed in protein-based films for the enhancement of mechanical properties [6]. Those phenolic compounds in the extract also acted as antioxidant and antimicrobial agents [7]. The seed coat of Bambara groundnut, typically removed before consumption or further processing, regarded as waste, has been exploited, especially as a promising source of phenolic compounds [4]. Klompong et al. [4] documented that the phenolic compounds extracted from Bambara groundnut seed coat with different types of media and extraction temperatures had total phenolic content in the range of 169–569 mg GAE/g dry extract, which were also showed varying antioxidative activities. However, no information concerning the impact of the extract from the seed coat of Bambara groundnut on properties of Bambara groundnut protein isolate-based film exists. The current study aimed to prepare and investigate the properties and characteristics of antioxidant film based on Bambara groundnut protein isolate incorporated with seed coat extract at varying concentrations.

## 2. Materials and Methods

### 2.1. Materials/Chemicals

Bambara groundnut seeds were bought from a local farm in Phatthalung province, Thailand. Ethanol was sourced from RCI Labscan Limited (Bangkok, Thailand), while glycerol was obtained from Ajax Finechem Pty. Ltd. (Taren Point, Australia). Sodium hydroxide (NaOH) was purchased from KemAus™ (Bangkok, Thailand). The rest of chemicals were acquired from Merck Ltd. in Thailand (Bangkok, Thailand).

### 2.2. Preparation of Bambara Groundnut Flour and Seed Coat

Bambara groundnut (BG) seeds were cleaned and immersed in water with a seed/water ratio of 1:10 (*w*/*v*) at 4 °C for 3 days for swelling of seed coat. Thereafter, the BG seed and the seed coat were separated manually. Both samples were further dried in a hot air oven (60 °C, 24 h). The dried seeds and seed coat were ground separately using high-speed blender for 1 min. The flour and seed coat powder were sieved through a 60-mesh screen. BG flour and seed coat powder were further used.

### 2.3. Extraction of Bambara Groundnut Protein Isolate (BG-PI)

BG flour was mixed with distilled water (DW) at a ratio of 1:50 (*w*/*v*). Then, the mixtures were adjusted to pH 11 with 2 M NaOH and stirred continuously at 28–30 °C (150 rpm, 2 h), followed by sedimentation at 4 °C for 12 h. Starch and other constituents were precipitated. The obtained supernatant rich in protein was subsequently adjusted to pH 4.5 with 2 M HCl acid and centrifuged (10,000× *g*, 5 min, 25 °C) to collect the protein. The isolated protein was freeze-dried at −50 °C for 72 h. BG-PI powder contained 1.57% moisture, 73.24% protein, 0.49% fat, 0.98% ash, and 23.72% carbohydrate as determined by the AOAC method [8]. BG-PI powder was kept in aluminium foil bags under vacuum condition and stored at −20 °C before use.

### 2.4. Preparation of BG Seed Coat Extract (BGSCE)

The BG seed coat powder was mixed with 80% (*v*/*v*) ethanol at a 1:15 (*w*/*v*) ratio [7] and ultrasonicated (70% amplitude, 20 min) by ultrasonic processor (Vibra-Cell™ VC 750, Sonics & Materials Inc., Newtown, CT, USA) using iced water as cooling medium during the process. Thereafter, the mixture was continuously stirred for 1 h and subsequently centrifuged (5000× *g*, 30 min) at 4 °C. The resulting supernatant was filtered using Whatman No. 1 filter paper, and the ethanol in filtrate was removed at 35 °C using an Eyela rotary evaporator. To remove any remaining ethanol, nitrogen gas was implemented for flushing. Thereafter, the dried extract was lyophilized at −50 °C for 72 h. BG seed coat extract powder was named ‘BGSCE’. The powder was stored in aluminium foil bags in vacuum condition as mentioned above.

### 2.5. Characterization of BGSCE

#### 2.5.1. Total Phenolic Content (TPC)

TPC of BGSCE was examined using Folin–Ciocalteu reagent (FCR). TPC was reported as mg gallic acid equivalent (GAE)/g dry extract [4].

#### 2.5.2. Antioxidative Activities

ABTS-RSA was measured, and DPPH-RSA was examined [7]. FRAP was assayed [9], and ORAC was also determined [7]. All activities were expressed as mmol Trolox equivalents (TE)/g dry extract.

#### 2.5.3. Active Compounds

Liquid chromatography/mass spectrometry (LC/MS) profiling and identification of BGSCE were conducted. The sample after dilution with deionized water was first separated using an Agilent 1100 series system (Agilent Technologies, Waldbronn, Germany) with a LiChroCART Purospher STAR RP-18e column (Merck, Branchburg, NJ, USA) (150 × 4.6 mm, i.d., 5 µm). Mobile phases A and B were acetonitrile and 10 mM ammonium formate buffer pH 4 adjusted with formic acid, respectively. The flow rate was 1.0 mL/min, and the temperature was 40 °C. The gradient program was as follows: 100% B constant (0–5 min), 0–20% A (5–10 min), 20% A constant (10–20 min), and 20–40% A (20–60 min). The detection was performed at wavelengths of 270, 330, 350, and 370 nm. Both positive and negative ionization modes were employed for MS detection, utilizing an electrospray ionization source with nitrogen gas as the drying gas. The operating parameters were set as 4000 V capillary voltage, 320 °C gas temperature, 13 L/min flow of the drying gas, and 60 psi nebulizer pressure. Quantitative analysis was performed using mass spectrometry detection (MSD) in SIM (Selected Ion Monitoring) mode.

### 2.6. Formation of Antioxidant Film Based on BG-PI Incorporated with BGSCE at Varying Concentrations

#### 2.6.1. Preparation of Film-Forming Solutions and Film Casting

BG-PI-based film was prepared as tailored by Nilsuwan et al. [10]. BG-PI powder was mixed with DW to achieve the protein concentration of 3.0% (*w*/*v*) and homogenized (5000× *g*, 1 min) to disperse powder. The mixtures were adjusted to pH 11 using 2 M NaOH and sonicated (28–30 °C, 10 min). Glycerol was added to BG-PI solutions at 50% (*w/w* based on protein content). BGSCE was incorporated into the prepared solutions at concentrations of 0 (control), 2, 4, 6, and 8% (*w*/*w* based on protein content). The mixtures were sonicated (28–30 °C, 10 min), adjusted to the final volume of 100 mL, and stirred for 1 h to dissolve BGSCE completely. All film-forming solutions (8.0 ± 0.1 g) were cast onto a rimmed silicone resin plate (5 cm × 5 cm). After air blowing (12 h, 25 ± 2 °C, 60 ± 5% RH, air velocity at 1.5 m/s), films were further dried in an environmental chamber (WTB Binder, Tuttlingen, Germany) at 25 ± 0.5 °C with 50 ± 5% RH for 24 h [10]. Dried films were further analyzed.

#### 2.6.2. Film Analyses

All films were cut and conditioned in an environmental chamber under the specified condition [10] for 48 h before testing.

##### Thickness

The thickness of films was measured using a micrometer. For each treatment, the thickness was measured at nine random locations on each of ten film samples.

##### Mechanical Properties

Films were cut into strips of fixed dimension of 2 cm × 5 cm. Tensile strength (TS), elongation at break (EAB), and elastic modulus (E) were determined and reported [7].

##### Water Vapor Permeability (WVP)

Films were placed over aluminum permeation cups filled with 0% RH dried silica gel and tightened with the aid of grease and rubber gasket [7]. After being placed in an environmental chamber (25 ± 2 °C, 50 ± 5% RH), the cup was weighed at 1 h intervals over a 10 h period. WVP was then computed as follows [7]:

(1) $$\mathrm{WVP}\;(\frac{g\;m}{m^{2}\;s\;\mathrm{Pa}})=\frac{\mathrm{wl}}{\mathrm{At}(P_{2}-P_{1})}$$

where *w* is weight gain (g), *l* is thickness of film (m), *A* is exposed film surface area (m^2^), *t* is time (s), and (*P*_2_ − *P*_1_) is vapor pressure difference across film (1583.7 Pa at 25 °C).

##### Water Contact Angle

Measurement of water contact angle was performed using a contact angle meter (Dataphysics GmbH, Filderstadt, Germany) equipped with image analysis software. The contact angle was assessed on both sides of films for five droplets at various locations. All measurements were conducted under controlled conditions (50 ± 5% RH, 25 ± 0.5 °C).

##### Color

Color measurements of the films were performed using a colorimeter (Hunterlab, Reston, VA, USA) as described by Tagrida, Nilsuwan, Gulzar, Prodpran, and Benjakul [7]. The parameters recorded included L*-, a*-, and b*-values. Total color difference (ΔE*) was computed as follows:

(2) $$\Delta E^{*}=\sqrt{{{(\Delta L}^{*})}^{2}+{\left({\Delta a}^{*}\right)}^{2}+{\left({\Delta b}^{*}\right)}^{2}}$$

where ΔL*, Δa*, and Δb* are the differences between the color parameter of the samples and those of the white standard (L* = 92.85, a* = −1.36, b* = 0.52).

##### Light Transmission and Opaqueness

Light transmission of the films in both the ultraviolet (200–280 nm) and visible (350–800 nm) ranges was determined [10] using UV–vis spectrophotometer (UV-1800, Shimadzu, Kyoto, Japan). The opaqueness value of the films was computed as follows:

(3) $$\mathrm{Opaqueness}\;\mathrm{value}=\frac{-\log T_{600}}{x}$$

where *T*_600_ is the fractional transmission at 600 nm and *x* is film thickness (mm).

##### Antioxidant Activities of Films

All films cut into small pieces (0.1 g) were mixed with distilled water (10 mL in a cap-tightened amber bottle). The mixture was shaken using an orbital shanker (200 rpm, room temperature, 24 h). After being centrifuged (20 min, 8000× *g*), the supernatants were determined for antioxidant activities as mentioned above [7]. All activities were expressed as μmol Trolox equivalent (TE)/g film.

##### Characterization of Films

All films were cut and conditioned in a desiccator with dried silica gel for 3 days before characterization.

- Fourier-transform infrared (FTIR) spectroscopy

FTIR spectra of the films were recorded using a horizontal ATR Trough plate crystal cell (45° ZnSe; 80 mm long, 10 mm wide and 4 mm thick) (PIKE Technology Inc., Madison, WI, USA) assembled to Bruker Model Equinox 55 FTIR spectrometer (Bruker Co., Ettlingen, Germany) at 25 °C. All obtained spectra were normalized before interpretation.

- Thermogravimetric analysis (TGA)

The thermostability of all films was assessed using a simultaneous thermal analyzer (STA-8000, PerkinElmer, Norwalk, CT, USA). Heating rate of 10 °C/min and temperature range of 30–800 °C were used. The thermal degradation temperature and weight loss of all film samples were recorded. Nitrogen as the purge gas was employed at a flow rate of 20 mL/min.

### 2.7. Statistical Analysis

Completely randomized design (CRD) was adopted for this study. Experiments and analyses were conducted in triplicate (n = 3). After analysis of variance (ANOVA) was performed, Duncan’s multiple range test was adopted for mean comparison at the *p*-value < 0.05. The analysis was carried out with a SPSS package (SPSS for windows, Version 28, SPSS Inc., Chicago, IL, USA).

## 3. Results and Discussion

### 3.1. Chemical Compositions and Antioxidant Activities of Bambara Groundnut Seed Coat Extract (BGSCE)

#### 3.1.1. TPC and Antioxidant Activities

The TPC of BGSCE was 708.38 mg GAE/g dry extract (Table 1). The TPC content was higher than those of groundnut seed coat extracts (169.23–567.18 mg GAE/g dry extract) extracted by three different solvents at various temperatures (30–90 °C) via the stirring process [4]. Ultrasonication could provide ultrasonic waves with high energy generated via microbubble explosion. The energy could facilitate the extraction of the solubilization of active compounds to the solvent [11]. In general, ethanol at an appropriate concentration could enhance the solubility or extractability of bioactive compounds from plant powder [12].

**Figure 1:**
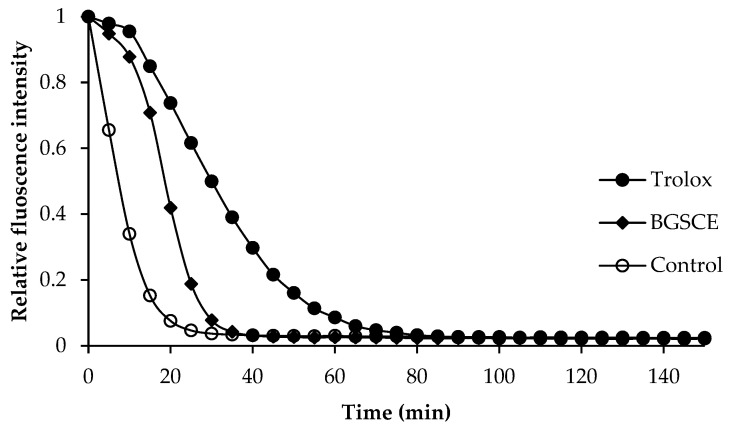
Fluorescence decay curves of Trolox (75 μM), BGSCE, and the control (without Trolox).

The antioxidant activities of BGSCE are shown in Table 1. The ABTS-RSA of BGSCE was 66.44 mmol TE/g dry extract, indicating its capacity to scavenge the stable radical cation ABTS•+, a blue-green chromophore with optimal absorbance at 734 nm. The intensity of this radical typically decreases in the presence of antioxidants [13]. BGSCE exhibited a DPPH-RSA of 4.98 mmol TE/g dry extract. DPPH serves as a free radical for evaluating antioxidant activity, which is largely attributed to the hydrogen-donating capacity of the test compounds [13]. DPPH activity is generally assessed using organic solvents like ethanol or methanol. This assay has a limitation in evaluating hydrophilic antioxidants, as DPPH• is soluble solely in organic media, particularly in alcohol, and not in aqueous solutions [13]. It is worth noting that the BGSCE extract had higher ability to donate a hydrogen atom or electron to the ABTS radical compared to the DPPH radical. This result might be related to the higher polarity of phenolic content in BGSC extract. The FRAP of BGSCE was 4.42 mmol TE/g dry extract. The FRAP assay is based on the reduction of ferric ions (Fe^3+^) to blue-colored ferrous (Fe^2+^) complex by antioxidants under acidic conditions [13]. Furthermore, BGSCE had an ORAC of 0.91 mmol TE/g dry extract. The ORAC assay evaluates the peroxyl radical scavenging capacity of antioxidants, which mitigate oxidation by transferring hydrogen atoms [7]. Fluorescence decay curves of fluorescein without and with 1 mg/mL BGSCE are depicted in Figure 1. The presence of BGSCE slowed down such a decay. BGSCE could therefore inhibit the reaction between peroxyl radicals and fluorescein, thereby preserving fluorescence. Hence, BGSCE exhibited antioxidant activity through various mechanisms, indicating its potential as a natural antioxidant for incorporation into protein isolate-based films.

#### 3.1.2. Phenolic Compounds Analyzed by LC/DAD/MSD

Several phenolic compounds of BGSCE are tabulated in Table 2. When negative mode was employed, higher sensitivity was gained than with positive mode [14]. As a negative ion is produced rapidly, LC/MS operating in the negative mode was effective for profiling the phenolic components [14]. Quercetin 3-galactoside was the most prevalent compound, followed by rutin, Azaleatin 3-arabinoside, Cinncassiol C3, and 6-C-Glucopyranosylepicatechin, respectively. Quercetin 3-galactoside (Hyperoside: Hyp), a flavonol glycoside, is known for its antioxidant properties [15]. The other identified compounds also demonstrated antioxidant activity [16,17]. It was observed that free phenolic acids were rarely detected, as they were often bound with sugar and presented as glycosides [18]. Nonetheless, phenolic compounds can be varied depending on genotype harvesting time, climate, maturity, soil conditions, extraction methods, and analytical techniques [19]. For positive mode, luteolin 6-C-glucoside 8-C-arabinoside (carlinoside) was dominant, followed by betavulgarin glucoside (Table 2). Luteolin 6-C-glucoside 8-C-arabinoside was also found in *Carlina vulgaris* flower and reported to exhibit antioxidant activity [20]. Therefore, BGSCE is considered a valuable source of antioxidants. Various phenolic compounds might contribute to the antioxidative activities of BGSCE (Table 2).

### 3.2. Properties and Characteristics of Films from Bambara Groundnut Protein Isolate (BG-PI) Containing BGSCE at Varying Concentrations

#### 3.2.1. Film Appearance and Thickness

Photographs of BG-PI-based film containing BGSCE at various concentrations are shown in Figure 1. A brownish color was observed for all films. Film without BGSCE had the lowest brownish color. Increasing brownish color was visually observed for the resulting films, as a higher concentration of BGSCE was incorporated. The highest brownish color was obtained for the film containing 8% BGSCE. Furthermore, the incorporation of BGSCE at higher concentrations resulted in a higher thickness of the resulting films (0.103–0.116 mm) (Table 3). BG-PI-based film without BGSCE (the control film) was thinner than those of films containing BGSCE at higher concentrations (6% and 8%) (*p* < 0.05). Thus, the incorporation of BGSCE rich in polyphenols increased the solid content. In addition, the interaction between polyphenols and protein chains in BG-PI could induce the entanglement of the matrix, leading to a relatively protruded network [21].

#### 3.2.2. Mechanical Properties

The TS and EAB of films made from BG-PI incorporated with varying concentrations of BGSCE are shown in Table 3. The control film had a TS of 0.44 MPa and EAB of 60.29%. With the incorporation of BGSCE, a continuous increase in TS was observed, and the highest TS (1.60 MPa) was reached for the film containing 8% BGSCE (*p* < 0.05). The addition of BGSCE enhanced the formation of junction zones within the film matrix through a protein–polyphenol interaction, resulting in a stronger film matrix [7,10,22]. This behavior is consistent with the improvement of the TS of soy protein isolate (SPI) films added with the addition of licorice residue extract (LRE) and chestnut bur extract (CBE) at 50 and 80 g/kg protein content, respectively, attributed to the protein–extract interactions [23,24]. The strength of the resulting BG-PI film with 4% BGSCE (1.09 MPa) was lower than those of SPI films containing 50 g/kg LRE (10.83 MPa) [23] and 80 g/kg CBE (2.1 MPa) [24], respectively. Moreover, the incorporation of BGSCE initially increased flexibility, as indicated by the rise in the EAB from 60.29% in the control film to 111.57% at 4% BGSCE. This result indicated that the addition of BGSCE at low-to-moderate concentrations (2–4%) could promote not only protein cross-linking but also plasticizing effects. Phenolic compounds might form cross-linking with protein chains to a moderate degree, which allowed for the protein chains to stretch under stress from mechanical testing. However, the addition of BGSCE at an excessive concentration (more than 4%) decreased the EAB of the resulting film, suggesting that the excessive cross-linking between the extract and protein chains might restrict the movement of the film matrix, leading to the augmented rigidity and lower capability of the stretch ability of film before breaking [7]. Similar results were found in the EAB of chicken protein isolate/fish skin gelatin (CPI/GE) films, which was increased as the tannic acid (TA) added was upsurged from 0.375 to 1.5% (*w*/*w*); however, it declined when a higher concentration of TA (3%) was used [10]. Furthermore, the elastic modulus (E) could support the increasing rigidity of the films with higher BGSCE concentrations. The control film had the lowest E value of 15.17 MPa, indicating a more flexible structure. As BGSCE concentrations were increased, the elastic modulus rose sharply to reach 101.02 MPa as 8% BGSCE was incorporated. This result indicated that the excessive cross-linking at higher phenolic concentrations could enhance the rigidity of the resulting film, which limited the capacity of the film to stretch. This was in alignment with the increase in TS and decrease in the EAB of film at higher concentrations (more than 4%) of BGSCE used.

#### 3.2.3. Water Vapor Permeability (WVP)

WVP is a crucial factor influencing the quality of food packaging materials. A low WVP is desirable food packaging, as it helps prevent water transfer between the food and the surrounding environment [25]. The WVP of BG-PI-based films was varied with the addition of BGSCE at different concentrations (Table 3). Film without BGSCE exhibited the highest WVP at 2.83 × 10^−10^ g m/m^2^ s Pa. A reduction in WVP (*p* < 0.05) was attained in the films containing BGSCE at higher concentrations. All films incorporated with BGSCE showed WVP values in the range of 1.76–2.45 × 10^−10^ g m/m^2^ s Pa. No difference between the WVP of films containing BGSCE at concentrations of 6% and 8% was observed (*p* > 0.05). Tagrida, Nilsuwan, Gulzar, Prodpran, and Benjakul [7] found a similar significant decrease in WVP (*p* < 0.05) for all gelatin/chitosan films when betel leaf ethanolic extract was incorporated. The reduction in WVP was achieved when BGSCE was added into the film. Han et al. [23] also found that the WVP values of SPI films were decreased from 2.04 × 10^−10^ to 1.64 × 10^−10^ g m/m^2^ s Pa when the concentration of LRE was elevated from 0 to 50 g/kg protein content. The increase in the interaction or cross-linking between phenolics and protein chains might decrease the distance between molecules in the film matrix, making less space or a denser structure for water vapor to pass through the film. Additionally, the reduction in the hydrophilicity of the film due to the interaction or cross-linking between protein chains and phenolic extracts could also cause lowered water vapor permeation through the resulting film [10,26]. Also, the denser network could serve as the barrier for water vapor migration. Furthermore, the optimal WVP value of BG-PI film containing 4% BGSCE (2.20 × 10^−10^ g m/m^2^ s Pa) was higher than SPI film containing 50 g/kg protein content LRE (1.64 × 10^−10^ g m/m^2^ s Pa) [23,24].

#### 3.2.4. Water Contact Angle

Film hydrophobicity was evaluated by measuring the water contact angle (*θ*_a(w))_ on the film surface (Table 3). Generally, an augmenting in *θ*_a(w)_ indicates a rise in the surface hydrophobicity of films [27]. A *θ*_a(w)_ greater than 60° signifies a more hydrophobic surface, whereas a *θ*_a(w)_ below 60° indicates more hydrophilic surface [28]. All films exhibited hydrophobic characteristics with *θ*_a(w)_ values ranging from 94.23° to 101.39°, suggesting their excellent water resistance. The *θ*_a(w)_ decreased with augmenting BGSCE concentrations in the films (*p* < 0.05). Polyphenols contain numerous polar groups such as −OH and = O, thus enhancing the wettability of the surface [29]. The wettability of the resulting film might also be affected by the rough surface of the tested films associated with the larger surface area. Moraczewski, Pawłowska, Stepczyńska, Malinowski, Kaczor, Budner, Gocman, and Rytlewski [29] documented that a film sample containing a higher amount of cocoa extract had the highest roughness with the lowest water contact angle value (62.4°), which was related with the highest wettability of this film. These findings demonstrated that an enhanced water wettability of the film as induced by BGSCE incorporation.

#### 3.2.5. Color

Visual observations indicated that homogenous films were produced regardless of the concentration of BGSCE used (Table 3). The incorporation of BGSCE resulted in a brownish color for the films, especially at great BGSCE concentrations. The film without BGSCE had the highest L* value (*p* < 0.05) but lowest a*-value and ΔE* value (*p* < 0.05) (Table 3). When BGSCE concentrations increased, the L* value of film decreased (*p* < 0.05), indicating a reduction in film lightness with a coincidental increase in redness. Conversely, the a*-value and ΔE* value upsurged (*p* < 0.05), suggesting the increases in both redness and total color change in the films containing BGSCE. Wang et al. [24] also documented that the reduction in the lightness (L*) and enhancement of redness (a*) and yellowness (b*) were observed in SPI films incorporated with CBE. This alteration was likely governed by pigmented compounds, particularly anthocyanins like procyanidin B1 and procyanidin B2 (Table 2). Similarly, Tagrida, Nilsuwan, Gulzar, Prodpran, and Benjakul [7] documented that the color change in the fish gelatin/chitosan blend films containing betel leaf ethanolic extracts was associated with the rising concentration of the extract, which contained some residual chlorophyll with a distinctive color. Consequently, the color difference of the films became more remarkable with the addition of BGSCE, especially at higher concentrations.

#### 3.2.6. Light Transmission and Opaqueness

**Figure 2:**
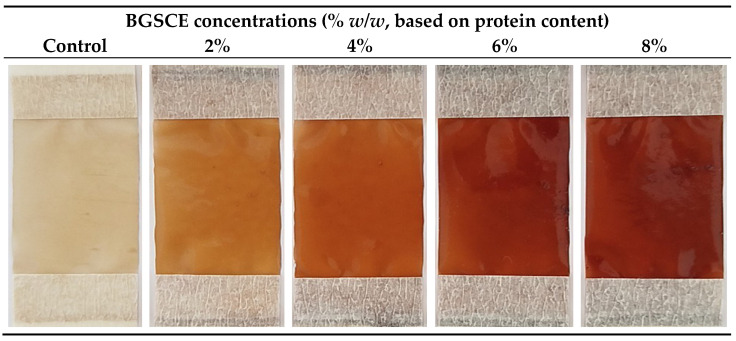
Appearance of films from Bambara groundnut protein isolate containing Bambara groundnut seed coat extract (BGSCE) at different concentrations. Control: film from Bambara groundnut protein isolate without BGSCE.

Films with effective UV–Vis light barrier abilities are desirable to prevent adverse quality changes [7]. The control film, which did not contain BGSCE, exhibited higher transmission (Table 4). It is worth noting that protein-based films possess excellent UV barrier properties [7]. UV has an adverse impact in food systems, particularly by enhancing lipid oxidation during the initial stage. Proteins containing amino acids, especially those with aromatic rings and double bonds, can absorb UV light [7]. The addition of BGSCE markedly enhanced the barrier abilities toward the UV and visible light of the resulting films (*p* < 0.05) compared to the control film. The film containing BGSCE had negligible transmission values in UV range (200–400 nm) regardless of the concentration of BGSCE used. The UV barrier ability of the film was related to the diverse phenolic compounds in BGSCE, such as quercetin 3-galactoside, rutin, and azaleatin 3-arabinoside (Table 2). Lower transmission was found in films containing BGSCE at higher concentrations (6% and 8%), indicating a greater capacity for polyphenols to act as a barrier to visible light. Additionally, visual observations (Figure 2) revealed that all prepared films with and without BGSCE were translucent, indicating their opaqueness. The opaqueness values upsurged with rising BGSCE concentrations (*p* < 0.05). Higher opaqueness values were associated with lower L* values. Therefore, the addition of BGSCE notably influenced the light transmission and opaqueness of the films to some extent. Similar behaviors were found for SPI films containing 0–70 g/kg LRE [23] and 0–100 g/kg CBE [24], respectively.

#### 3.2.7. Antioxidant Activities of Films Containing BGSCE at Different Concentrations

Antioxidant activities of films from Bambara groundnut protein isolate incorporated with BGSCE at different concentrations were evaluated using various assays (Table 5). The control film without BGSCE showed a low ABTS-RSA and FRAP, while no DPPH-RSA was detected. The side chains of protein isolate molecules, particularly the amine and hydroxyl groups, could play a role in radical scavenging activities [23]. The ABTS-RSA, DPPH-RSA, FRAP, and ORAC of the resulting films increased with augmenting concentrations of BGSCE from 2% to 8% (*p* < 0.05). This increase was associated with the presence of polyphenols and other reducing compounds such as terpenoids and alkaloids in BGSCE released from the films [23], which might function as antioxidants. They served as electron donors or free radical acceptors, facilitating the reduction in ferric ions to ferrous ions. They also possessed peroxyl radical scavenging activity [13]. Likewise, the addition of licorice ethanolic extract to soy protein isolate films resulted in enhanced antioxidant activities [23]. Additionally, Kevij*,* et al. [30] discovered an increase in antioxidant activities of whey protein isolate-based films when the curcumin concentration increased.

#### 3.2.8. Fourier-Transform Infrared (FTIR) Spectroscopy

**Figure 3:**
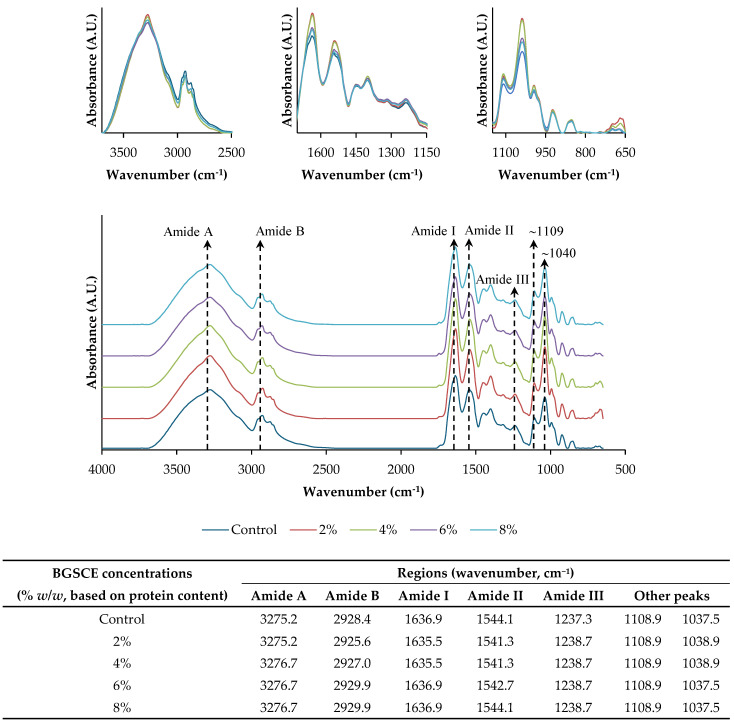
ATR-FTIR spectra of films from Bambara groundnut protein isolate containing Bambara groundnut seed coat extract (BGSCE) at different concentrations.

The FTIR spectra of all samples (Figure 3) displayed a peak around 3275.2–3276.7 cm^−1^ (amide A), representing the inter- and intramolecular H-bonding as well as O–H and N–H stretching vibrations [30]. The increase in intensity of this peak in films containing BGSCE was attributed to –OH groups present in polyphenols of BGSCE. Additionally, films with BGSCE exhibited a slight shift of the amide A band to a higher wavenumber. Wang et al. [24] documented that soy protein isolate films containing chestnut bur extract exhibited a minor shift of the amide A band to higher wavenumbers (3270–3274 cm^−1^), which may be attributed to reduced hydrogen bonding. This shift could be associated with the formation of other types of bonds, particularly hydrophobic interactions between the protein and phenolic molecules [24]. Peaks observed at wavenumbers ranging from 2928.4 cm^−1^ to 2929.9 cm^−1^ were mediated by the aliphatic C-H stretching vibrations associated with the CH_3_ and CH_2_ functional groups (amide B) [24]. Peaks observed at wavenumbers of 1639.2, 1537.4, and 1255.0 cm^−1^ corresponded to the C=O stretching (amide I), N–H bending with C–N groups stretching vibrations (amide II), and vibrations in plane of C–N and N–H (amide III), respectively [30]. The wavenumbers of amide I and II of films containing 2% and 4% BGSCE slightly shifted to lower wavenumbers, indicating an interaction between the protein and BGSCE molecules. Moreover, an increase in the peak intensity at amide I, II, and III mostly implied that the polyphenol–protein interaction through the hydrogen bond was weakened as BGSCE at greater concentrations was added. This result was similar to Wang, Hu, Ma, and Wang [24], who also documented that the addition of 10% CBE increased the peak intensity at the wavenumbers of 3274 cm^−1^, 1625 cm^−1^ (amide I), and 1535 cm^−1^ (amide II). Higher intensity was observed for peaks at the wavenumber of 1108.9 cm^−1^ and 1037.5–1038.9 cm^−1^ of all films containing BGSCE, corresponding to the C–OH stretch and C–O stretching vibration [22,23], respectively. Those were mainly associated with the presence of plasticizers such as glycerol and phenolic compounds [23], which were observed for all films containing BGSCE. Overall, the addition of BGSCE had an influence on the FTIR spectra of the resulting films. Some shift of peaks at wavenumbers of 1635.5–1636.9 cm^−1^ and 1541.3–1544.1 cm^−1^ indicated the interaction between polyphenols and protein isolate.

#### 3.2.9. Thermogravimetric Analysis (TGA)

**Figure 4:**
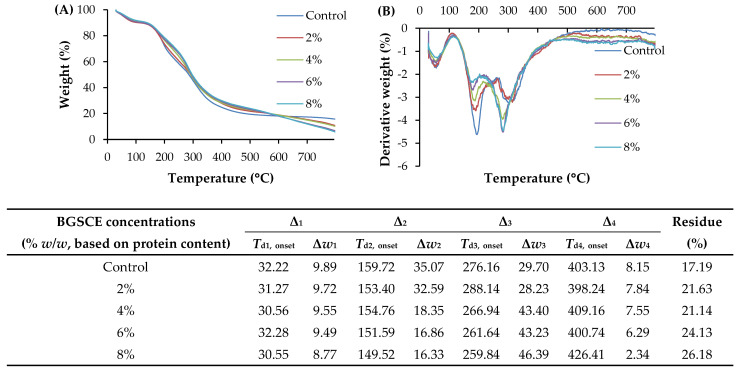
The thermo-gravimetric curves (**A**,**B**), degradation temperatures (*T*_d_, °C), and weight loss (Δ*w*, %) of films from Bambara groundnut protein isolate containing Bambara groundnut seed coat extract (BGSCE) at different concentrations. Δ_1_, Δ_2_, Δ_3_, and Δ_4_ denote the first, second, third, and fourth stages of the weight loss of films during a heating scan, respectively.

Thermograms representing the thermal degradation behavior of all films are depicted in Figure 4. Four main stages of weight loss were found in all samples. The first stage weight loss (Δ_1_) at Δ*w*_1_ in the range of 8.77–9.89% was obtained with onset degradation temperatures (*T*_d1,onset_) of 30.55–32.22 °C. The weight loss was an indicator of the loss of free water and the release of other volatile compounds absorbed in the film [7]. It was observed that the film containing 8% BGSCE exhibited lower weight loss, indicating reduced water absorption in the film matrix compared to the control film. Tagrida, Nilsuwan, Gulzar, Prodpran, and Benjakul [7] reported that the fish gelatin/chitosan (G7.5:C2.5) blend films incorporated with 2% betel leaf ethanolic extracts also had lower weight loss during the first stage. The second stage (Δ_2_) of weight loss (Δ*w*_2_ = 16.33–35.07%) appeared at *T*_d2,onset_ of 149.52–159.72 °C. This stage is associated with the degradation of the film matrix, particularly the loss of short chain and looser polymeric molecules as well as the plasticizer (glycerol) [31]. For the third stage (Δ_3_) of weight loss, a Δ*w*_3_ of 28.23–46.39% and *T*_d3,onset_ of 259.84–288.14 °C were observed in all film samples, primarily linked to the degradation of larger-sized or aggregated proteins. Lower degradation temperatures (*T*_d3,onset_) with higher weight loss (Δ*w*_3_) were generally noticed in films containing BGSCE, especially at high concentrations used (more than 4%). This might be connected to a less ordered and compact network compared to the control film without BGSCE. This finding aligned with the mechanical properties. All films had the fourth stage (Δ_4_) of weight loss at Δ*w*_4_ in the range of 2.34–8.15% and *T*_d4,onset_ in the range of 398.24–426.41 °C. The fourth stage of weight loss took place at higher temperatures due to the breaking of S-S, O-N, and O-O linkages of proteins [32]. Lower Δ*w*_4_ was attained for all films containing BGSCE, compared to that of the control film regardless of the concentration of BGSCE used. This might be in accordance with a less ordered structure of the film containing BGSCE. Nevertheless, the higher residual mass or char content (21.63–26.18%) remained after heating was observed for all films containing BGSCE than that of the control sample (17.19%).

## 4. Conclusions

Bambara groundnut seed coat extract (BGSCE) possessed high total phenolic content and antioxidant activities with different modes of action, including ABTS and DPPH radical scavenging activities, ferric reducing antioxidant power (FRAP), and oxygen radical absorbance capacity (ORAC). The concentrations of BGSCE added had an impact on the properties and characteristics of Bambara groundnut protein isolate (BG-PI)-based films. BGSCE enhanced the thickness, tensile strength, water vapor barrier, redness, UV-light barrier, and antioxidant activities of films depending on the concentrations used. FTIR revealed an increase in hydroxyl groups and interactions through hydrogen bonding in the film matrix when BGSCE at high concentrations was incorporated. Films containing BGSCE had lower thermal stability. Therefore, the active film from BG-PI containing 4% BGSCE with good mechanical properties and bioactivities could be further used as active packaging for quality maintenance of food products prone to lipid oxidation.

## Figures and Tables

**Table 1:** Total phenolic content and antioxidant activities of Bambara groundnut seed coat extract (BGSCE).

Parameters	BGSCE *
Total phenolic content (TPC) (mg GAE/g dry extract)	708.38 ± 73.29
ABTS radical scavenging activity (mmol TE/g dry extract)	66.44 ± 1.71
DPPH radical scavenging activity (mmol TE/g dry extract)	4.98 ± 0.10
Ferric reducing antioxidant power (FRAP) (mmol TE/g dry extract)	4.42 ± 0.15
Oxygen radical absorbance capacity (ORAC) (mmol TE/g dry extract)	0.91 ± 0.05

* Values are expressed as mean ± SD (n = 3). GAE: Gallic acid equivalent; TE: Trolox equivalent.

**Table 2:** Phenolic compounds in Bambara groundnut seed coat extract analyzed by LC/DAD/MSD in negative and positive modes.

Identified Compounds	Formula	Mass	R_t_ (min)	Abundance (×10^5^)
**Negative mode**				
Quercetin 3-galactoside	C_21_H_20_O_13_	465.1	13.0	178.3
Rutin	C_27_H_30_O_16_	610.2	11.9	104.4
Azaleatin 3-arabinoside	C_21_H_20_O_12_	448.1	14.7	87.8
Cinncassiol C3	C_20_H_30_O_7_	382.2	12.1	41.8
6-C-Glucopyranosylepicatechin	C_21_H_24_O_11_	452.1	8.2	38.4
Isoscoparin 2′-O-glucoside	C_28_H_32_O_17_	624.2	12.4	28.4
Quercetin 3-glucosyl-(1->2)-[rhamnosyl-(1->6)-galactoside]	C_33_H_40_O_21_	772.2	11.3	21.9
Procyanidin B2	C_30_H_26_O_12_	578.1	9.7	21.7
Quercetin 3-methyl ether 3′-xyloside	C_21_H_20_O_11_	448.1	15.0	18.1
(±)-Catechin	C_15_H_14_O_7_	290.1	10.4	12.6
Soyasaponin bg	C_54_H_84_O_21_	1068.5	20.9	11.9
Pyrocatechol	C_6_H_6_O_2_	110.0	6.4	11.9
5,7,3′,4′-Tetrahydroxy-4-phenylcoumarin 5-O-apiosyl-(1->6)-glucoside	C_26_H_28_O_15_	580.1	11.9	11.4
Theogallin	C_14_H_16_O_10_	344.1	4.6	10.9
Quercetin	C_15_H_10_O_7_	302.0	16.6	8.7
Apigenin 7-O-glucoside	C_21_H_20_O_10_	432.1	12.2	8.7
Cinncassiol D2 glucoside	C_26_H_42_O_11_	530.3	14.0	7.0
Phenyl glucuronide	C_12_H_14_O_7_	270.1	6.8	5.8
4-Glucogallic acid	C_13_H_16_O_10_	332.1	2.3	4.7
Procyanidin B1	C_30_H_26_O_12_	578.1	9.9	3.9
Momordin IIa	C_48_H_76_O_18_	940.5	20.6	3.8
Luteolin 3′-methyl ether 4′-glucoside	C_22_H_22_O_11_	462.1	16.2	3.4
Gallic acid	C_7_H_6_O_5_	170.0	3.1	3.2
Methyl 2,4,6-trihydroxybenzoate	C_8_H_8_O_5_	184.0	2.9	3.2
Methyl 6-O-galloyl-beta-D-glucopyranoside	C_14_H_18_O_10_	346.1	1.7	1.7
**Positive mode**				
Luteolin 6-C-glucoside 8-C-arabinoside	C_27_H_30_O_16_	610.2	11.7	41.1
Betavulgarin glucoside	C_23_H_22_O_11_	474.1	8.1	8.9
Boviquinone 4	C_26_H_36_O_4_	412.3	37.9	6.3
[12]-Gingerdione	C_23_H_36_O_4_	376.3	32.0	3.5
Bolegrevilol	C_28_H_40_O_4_	440.3	39.4	3.3
(13R,14R)-7-Labdene-13,14,15-triol	C_20_H_36_O_3_	324.3	34.8	1.9
[7]-Paradol	C_18_H_28_O_3_	292.2	23.7	1.8

**Table 3:** Thickness, mechanical properties, water vapor permeability, and color of films from Bambara groundnut protein isolate containing Bambara groundnut seed coat extract (BGSCE) at different concentrations.

Parameters	BGSCE Concentrations (% *w*/*w*, Based on Protein Content)				
	Control	2%	4%	6%	8%
**Film thickness (mm)**	0.103 ± 0.008 b *	0.105 ± 0.005 b	0.102 ± 0.005 b	0.114 ± 0.007 a	0.116 ± 0.008 a
**Tensile strength (MPa)**	0.44 ± 0.05 e	0.72 ± 0.07 d	1.09 ± 0.15 c	1.43 ± 0.34 b	1.60 ± 0.15 a
**Elongation at break (%)**	60.29 ± 7.05 b	112.50 ± 11.19 a	111.57 ± 11.94 a	56.69 ± 14.50 b	26.33 ± 7.16 c
**Elastic modulus (MPa)**	15.17 ± 5.01 d	22.85 ± 4.62 cd	32.91 ± 6.16 c	79.06 ± 27.31 b	101.02 ± 15.61 a
**Water vapor permeability** **(×10^−10^ g m/m^2^ s Pa)**	2.83 ± 0.09 a	2.45 ± 0.10 b	2.20 ± 0.13 c	1.85 ± 0.16 d	1.76 ± 0.11 d
**Water contact angle (°)**	101.39 ± 0.31 a	99.35 ± 0.52 b	98.54 ± 0.14 c	97.52 ± 0.24 d	94.23 ± 0.31 e
L*	72.13 ± 0.60 a	53.36 ± 0.31 b	43.86 ± 0.77 c	36.44 ± 0.80 d	35.10 ± 1.35 e
a*	3.49 ± 0.25 d	17.88 ± 0.11 c	23.90 ± 0.20 a	22.02 ± 1.26 b	21.02 ± 0.97 b
b*	25.31 ± 0.23 b	37.53 ± 0.95 a	36.04 ± 0.89 a	21.01 ± 1.27 c	17.22 ± 1.63 d
ΔE*	32.67 ± 0.25 d	57.44 ± 0.49 c	65.58 ± 0.18 a	64.43 ± 0.15 b	64.18 ± 0.57 b

* Values are expressed as mean ± SD (n = 3). Different lowercase letters in the same row denote significant difference (*p* < 0.05).

**Table 4:** Light transmission and opaqueness of films from Bambara groundnut protein isolate containing Bambara groundnut seed coat extract (BGSCE) at different concentrations.

Parameters		BGSCE Concentrations (% *w*/*w*, Based on Protein Content)				
		Control	2%	4%	6%	8%
**Light transmission (%)** **at different wavelength** **(nm)**	200	0.00 ± 0.00 *	0.00 ± 0.00	0.00 ± 0.00	0.00 ± 0.00	0.00 ± 0.00
	280	0.00 ± 0.00	0.00 ± 0.00	0.00 ± 0.00	0.00 ± 0.00	0.00 ± 0.00
	200	5.79 ± 0.37 a	0.64 ± 0.18 b	0.05 ± 0.10 c	0.00 ± 0.00 c	0.00 ± 0.00 c
	400	15.45 ± 0.76 a	4.53 ± 0.40 b	2.43 ± 0.45 c	0.14 ± 0.04 d	0.10 ± 0.03 d
	500	30.23 ± 1.73 a	12.51 ± 0.90 b	8.37 ± 0.59 c	0.88 ± 0.07 d	0.70 ± 0.07 d
	600	39.68 ± 1.94 a	23.52 ± 1.61 b	20.07 ± 0.80 c	3.10 ± 0.40 d	2.83 ± 0.11 d
	700	46.32 ± 2.36 a	33.27 ± 1.84 b	31.51 ± 2.08 b	5.24 ± 0.71 c	5.51 ± 0.46 c
	800	50.24 ± 2.64 a	37.59 ± 1.80 b	37.21 ± 2.60 b	6.17 ± 1.02 c	6.58 ± 0.62 c
**Opaqueness**		3.80 ± 0.12 d	5.93 ± 0.29 c	6.87 ± 0.36 b	13.17 ± 0.80 a	13.30 ± 0.58 a

* Values are expressed as mean ± SD (n = 3). Different lowercase letters in the same row denote significant difference (*p* < 0.05).

**Table 5:** Antioxidant activities of films from Bambara groundnut protein isolate containing Bambara groundnut seed coat extract (BGSCE) at different concentrations.

Parameters	BGSCE Concentrations (% *w*/*w*, Based on Protein Content)				
	Control	2%	4%	6%	8%
**ABTS radical scavenging activity** **(μmol TE/g dry extract)**	44.29 ± 3.14 a *	114.20 ± 4.36 b	138.20 ± 21.70 c	285.33 ± 5.77 d	358.67 ± 5.77 e
**DPPH radical scavenging activity** **(μmol TE/g dry extract)**	ND	7.01 ± 0.33 b	8.88 ± 0.46 b	8.88 ± 1.12 b	12.19 ± 1.47 a
**FRAP (μmol TE/g dry extract)**	1.06 ± 0.15 d	6.96 ± 1.25 c	8.80 ± 1.53 c	17.33 ± 1.18 b	28.61 ± 9.12 a
**ORAC (μmol TE/g dry extract)**	66.93 ± 4.45 d	81.49 ± 0.65 c	92.82 ± 6.7 b	97.97 ± 10.25 b	110.26 ± 3.31 a

* Values are expressed as mean ± SD (n = 3). Different lowercase letters in the same row denote significant difference (*p* < 0.05). TE: Trolox equivalent. ND: Not detected.

## Data Availability

The original contributions presented in the study are included in the article, further inquiries can be directed to the corresponding author.
